# Flow Injection Analysis with Direct UV Detection Following Electric Field Driven Membrane Extraction

**DOI:** 10.3390/molecules23051000

**Published:** 2018-04-25

**Authors:** Hong Heng See, Nor Akma Mamat, Peter C. Hauser

**Affiliations:** 1 Centre for Sustainable Nanomaterials, Ibnu Sina Institute for Scientific and Industrial Research, Universiti Teknologi Malaysia, 81310, Johor Bahru, Johor, Malaysia;norakma_0689@yahoo.com; 2 Department of Chemistry, Faculty of Science, Universiti Teknologi Malaysia, 81310, Johor Bahru, Johor, Malaysia; 3 Department of Chemistry, University of Basel, Spitalstrasse 51, 4056 Basel, Switzerland; Peter.Hauser@unibas.ch

**Keywords:** electromembrane extraction, matrix elimination, miniaturization, flow-injection analysis

## Abstract

A method for on-line matrix elimination to enable selective quantification of ultraviolet absorbing analytes by a flow-injection analysis procedure is described. Selectivity is achieved by electric field driven extraction across a polymer inclusion membrane. The method was demonstrated on the example of the determination of naproxen from spiked human urine. Membranes of 10 μm thickness were employed which consisted of 7.5 mg cellulose triacetate as base polymer, 5 mg of *o*-nitrophenyl octyl ether as plasticizer and 7.5 mg of Aliquat 336 as cationic carrier. Ten μL of sample was introduced into a continuous stream of background solution consisting of 100 µM aqueous NaClO_4_ with a flow rate of 2 μL/min while applying a voltage of 150 V to the extraction cell. The target ion was electrokinetically transported across the membrane and enriched in 1.5 μL of a stagnant acceptor solution. This was subsequently pumped past a flow-through UV detector for quantification. The method showed a linear range from 5 to 200 µM with a correlation coefficient of 0.9978 and a reproducibility of typically 7% (*n* = 8). The detection limit of the method for naproxen was 2 µM.

## 1. Introduction 

Many analytical methods dealing with samples with complex matrices require a pretreatment step for matrix removal prior to quantification by instrumental measurement. Liquid-liquid extraction (LLE) is the traditional approach which has now to a large extent been replaced by solid phase extraction (SPE). Disadvantages of both variants are the fact that they require at least two steps and their need for organic solvents. Recently, several microscale methods have been introduced to speed up and simplify the procedures as well as to minimize organic solvent usage [1,2,3,4,5]. One of the new approaches involves the application of a voltage to enhance liquid-phase extraction of charged analytes from aqueous samples. The use of an electric field as driving force imparts a degree of selectivity in that only ions of correct charge (positive or negative) are transported according to the polarity of the applied field and neutral species are excluded. At least in principle, faster extractions might also be achieved when not relying on passive diffusion. This technique, usually termed ‘electromembrane extraction’ (EME), has been based on supported liquid membranes (SLM) consisting of a porous polymeric membrane impregnated with a water-immiscible organic solvent [6]. This procedure has been intensively explored and found to be applicable to various charged species [7,8,9]. Nevertheless, as the SLMs have limited mechanical stability due to the leaching of the liquid-phase from the membrane [10,11], the use of polymer inclusion membranes (PIM) as an alternative for electric field driven membrane extraction was recently investigated by us [12,13,14,15,16,17]. PIMs are homogeneous, self-supporting plasticized polymeric membranes, which for these investigations consisted of cellulose triacetate as base polymer, a plasticizer, and usually an ion carrier. This robust material was proven to be suitable for the extraction of lipophilic organic ions [12,13], including pesticides [15] as well as inorganic ions [14]. 

Flow injection analysis (FIA) is a well-established technique for automation of analysis based on injecting a sample into a flowing carrier stream. The selectivity for quantification is usually achieved either by employing a selective reagent, which leads to a characteristic coloured product, or by employing a selective flow through detector. The integration of liquid phase extraction into flow injection analysis was first reported by Karlberg and Thelande in 1978 [18]. Their system was based on a two-phase flow (alternating segments of aqueous donor and organic acceptor) and required a phase separator before the detector flow through cell. Advanced and more robust diffusion based liquid phase extraction systems based on membranes to separate donor and acceptor streams were later reported; for instance, designs based on a microporous membrane [19], a SLM [20], and a PIM [21] were successfully integrated into FIA.

The use of EME in the FIA approach is demonstrated for the determination of naproxen in human urine samples. Naproxen, (S)-6-methoxy-α-methyl-2-naphthaleneacetic acid, is a non-steroidal anti-inflammatory drug (NSAID) commonly used for the treatment of pain, fever, inflammation, and stiffness [22]. It is classified as the least harmful NSAID for chronic use for people with high risk of cardiovascular complications [23]. On the other hand, one of the potential side effects of excessive or long-term use of naproxen is an increase in the risk of stomach ulceration [24]. After oral administration, naproxen is partially metabolized to its 6-*O*-desmethylated metabolite and then free naproxen is excreted in urine together with metabolite [25]. Determination of NSAIDs including naproxen in aqueous matrices has been attempted commonly using chromatographic methods coupled with various detectors. Moreover, various sample preparation methods have been investigated, with most of them based on SPE [26,27], liquid phase microextraction [28], solid phase microextraction [25,29], and EME [30] techniques. It has a strong absorbance band in the ultraviolet (UV), which makes it accessible for quantification by molecular absorption spectrometry following matrix elimination.

## 2. Results and Discussion

### 2.1. System Design and Operation

**Figure 1:**
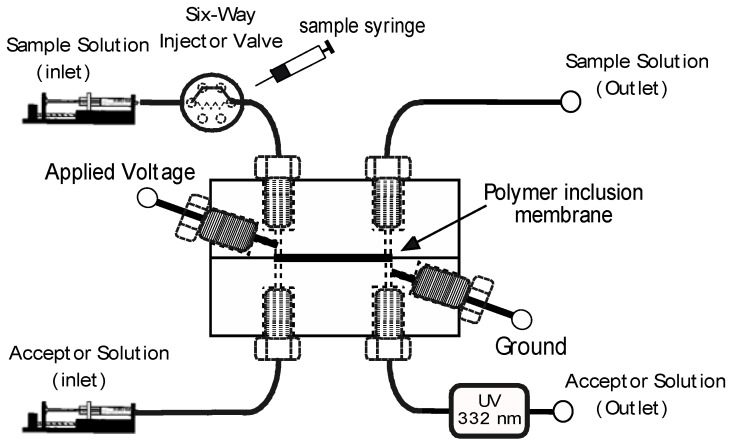
Schematic drawing of the electromembrane extraction (EME)-cell and the associated manifold including the UV-absorption detector. Not drawn to scale.

A schematic diagram of the system is shown in Figure 1. The EME-cell is a miniaturized version of a thin layer cell reported previously by us [13], with now a volume of as little as 1.5 µL for both, donor and acceptor chambers. This is matched to the on-capillary UV-absorption detector (borrowed from capillary electrophoresis) used in this project. The cell is incorporated into a manifold which is based on two programmable syringe pumps to drive solutions and a rotary valve for injection of samples. The volumes of donor and acceptor solutions moved through the manifold are fully controlled by the programmable syringe pumps which are able to provide flow rates in the range of 0.1 µL/min to 50 µL/min.

An overview of a typical series of operations is given in Table 1. The movement of the syringe pump is controlled by presetting the desired flow rate. The protocol starts with flushing of the donor and acceptor cell channels with a background electrolyte solution for approximately 2 min. The pump attached to the acceptor side of the cell is then turned off to keep the acceptor solution stagnant during the extraction. The extraction voltage is then turned on and the sample preloaded in the sample loop of the injection valve (10 µL) passed through the extraction cell by continuous streaming. Following the extraction, the acceptor side pump is turned on again in order to pass the analyte plug through the detector for quantification. Note that in contrast to classical FIA, the stream on the receiving side of the cell is not continuously flowing. This is in the interest of maintaining a sharp segment high in analyte concentration. The entire procedure, including purging of the manifold, requires approximately 10 min to complete. 

**Figure 2:**
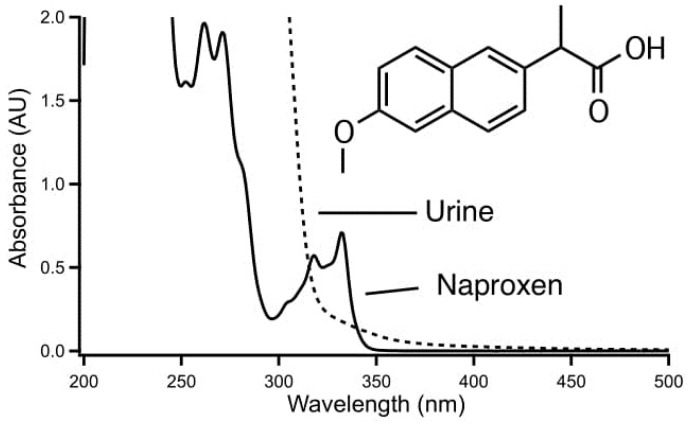
Absorbance spectra of naproxen (10 mg/100 mL methanol) and urine (diluted 1:3 (*v*/*v*) with methanol and then centrifuged).

The utility of the proposed approach is illustrated by the spectra of Figure 2 for the model analysis chosen to demonstrate the method. Naproxen shows strong absorbance below 300 nm as seen in Figure 2A. Substances such as the analgesics acetaminophen, phenacetin, and salicylic acid, which possibly may be found in urine, are showing strong absorbance at the same range. However, in contrast to these compounds, naproxen also has an absorbance band at the relatively long wavelength of 330 nm. This is due to the fact that it has two fused aromatic rings, and this additional band imparts a certain degree of selectivity in the determination by molecular absorption spectrometry as compounds of this nature are relatively rare. Direct determination in urine on the other hand is not possible since this matrix shows a background absorption up to about 400 nm as shown in Figure 2B for a blank urine sample not containing any drugs. While the 330 nm band of naproxen is located on the edge of the background absorption of urine this cannot be expected to be uniform between samples. Note that in Figure 2 the intensity of the absorption of naproxen relative to the urine background is not representative for a real sample. The proposed procedure imparts a degree of selectivity as it discriminates against species of the wrong charge, species of low lipophilicity, and neutral species, and will be suitable for this application if it allows the separation of naproxen without also extracting the components from the sample matrix responsible for the background absorption. 

### 2.2. Membrane Composition and Operating Conditions

**Figure 3:**
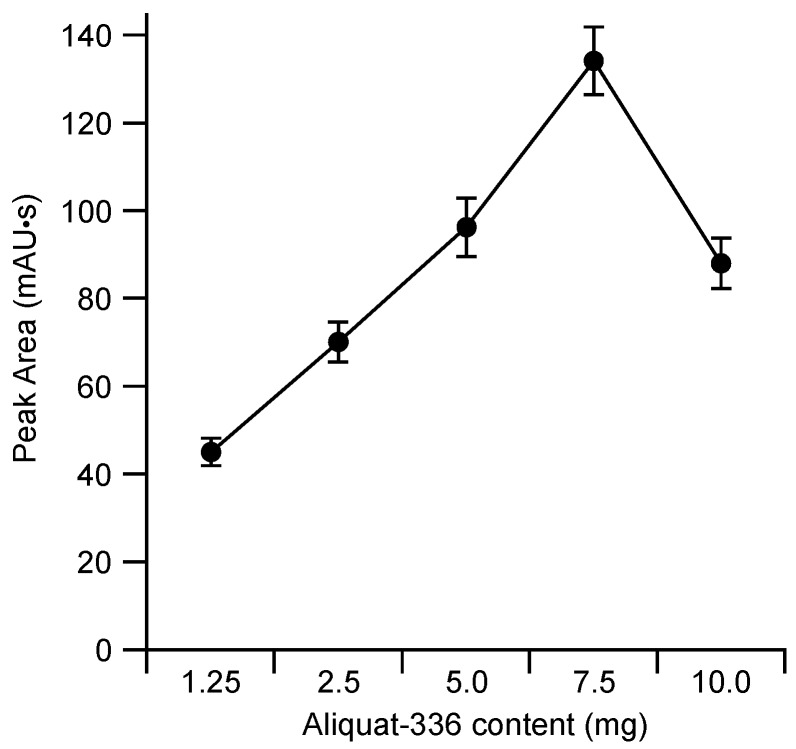
Effect of the content of Aliquat 336 in membranes based on 7.5 mg cellulose triacetate (CTA) and 5 mg *o*-nitrophenyl octyl ether (NPOE) on the extraction efficiency of naproxen. Donor background: 100 μM NaClO_4_ at 2 μL/min; acceptor phase: 100 μM NaClO_4_ in 1.5 µL; standard: 10 μL of 50 μM naproxen in 100 μM NaClO_4_, applied voltage: 150 V. Each data point represents the mean value for five measurements.

The constitution of the membrane employed for the electric field driven extraction was based on our previous experience with plasticized polymeric membranes [12,13,14,15]. In a comparison between poly(vinyl chloride) and cellulose triacetate based membranes it had been found that only the latter was suitable as base material since the rate of transport through the former was inadequate [12]. Also, the thinner the membrane, the faster the rate of transport [15]. The thickness of the cellulose triacetate-based membranes tested in this project was between about 10 and 25 µm, which is at the minimum required for sufficient mechanical robustness. Due to its flexibility, the thinnest membrane could in fact only be employed as the new cell used for this work had a narrower channel than the previous cell (a width of 1 mm instead of 4 mm). The effects of the nature and amount of plasticizer in the membrane are not as clear-cut and depend on the nature of the species to be transported [13,14]. As a fully comprehensive optimization in this regard is a tedious procedure a fixed proportion of *o*-nitrophenyl octyl ether (NPOE) in the membrane composition was employed, which based on our previous experience was expected to give generally good results. Similarly, the proportion of the cationic carrier Aliquat 336 in the membrane has an effect on the transport efficiency, which is also different for different target species in that it was found that for less lipophilic anions the maximum was found for higher proportions of carrier than for the more lipophilic anions [13]. The results shown in Figure 3 demonstrate that also for naproxen a strong dependence on the carrier content in the membrane was obtained. The optimum composition according to the plot of Figure 3, i.e., a membrane consisting of 7.5 mg cellulose triacetate (CTA), 5 mg NPOE, and 7.5 mg Aliquat 336, was adopted for the further investigations. Note that the carrier contents around the optimum values were also tested and no significant improvement was observed.

**Figure 4:**
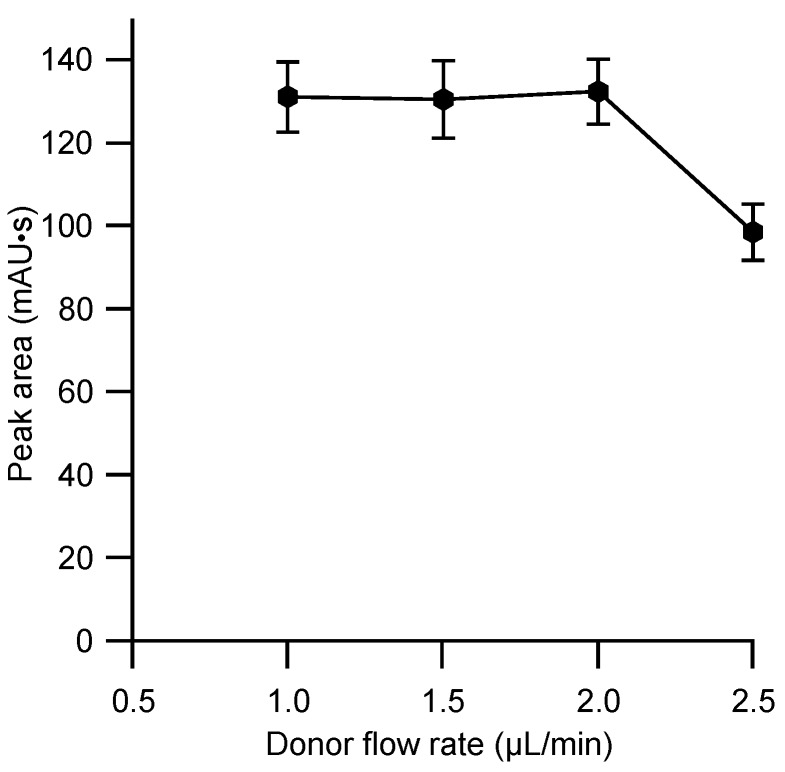
Effect of donor background flow rate on the extraction efficiency of naproxen. Membrane: CTA 7.5 mg, NPOE 5 mg, Aliquat 336 7.5 mg. Other conditions as for Figure 3. Each data point represents the mean value for five measurements.

The rate of extraction is generally proportional to the voltage applied to the cell [15] and the highest possible voltage for stable operation with the cell employed of 150 V was adopted. The sample introduced via the injector valve is passed through the donor channel in the extraction cell with a continuous steady flow of background electrolyte. The residence time of the sample plug containing the naproxen at the sample–membrane-interface is therefore determined by the flow rate and it was expected that the latter will have a strong effect on the extraction efficiency. This aspect was therefore investigated, and the results are given in Figure 4. A maximum was obtained for the lower flow rates, but this dropped above 2 µL/min as illustrated in Figure 4. As the flow rate is inverse to analysis time, the highest possible flow rate without penalty in efficiency, 2 µL/min, was therefore adopted. The same flow rate was adopted for the subsequent transport of the acceptor plug through the detector. This showed largely the expected inverse effect of the flow rate on the peak width, but at higher flow rates tested (3 µL/min and 4 µL/min), an undesired reduction of the peak height was observed, indicating a dispersion.

### 2.3.Method Validation

A series of experiments to determine linearity, limits of detection (LOD), and the method’s reproducibility was performed. Calibration curves were acquired by using optimized extraction conditions for aqueous standard solutions at eight concentration levels in the range from 1 μM to 200 μM. The curves of UV response peak area (mAU·s) versus analyte concentration (μM) were found to be linear in the range of 5 μM to 200 μM with a good correlation coefficient of 0.9978. The limit of detection and quantification of naproxen were determined at a signal-to-noise ratio of 3 and 10 and were found to be 2 μM and 7 μM, respectively. The method’s reproducibility was determined for 50 μM and 200 μM with eight consecutive experiments performed at each level and the peak areas could be reproduced to within 6.3% and 7.5% relative standard deviation.

### 2.4. Spiked Urine Sample

The method was then tested for its suitability for the determination of naproxen in urine. Naproxen was spiked at two different concentrations (50 μM and 200 μM) into drug-free urine and eight consecutive analyses performed at each level using the same membrane. The results were evaluated by comparison with the calibration curve obtained from the aqueous standards. The relative recoveries for the method are given in Table 2 (note that these denote the ability of the method for correct quantification of naproxen in urine and are not the extraction efficiencies of the EME cell). Both the recoveries and the precision values also given in Table 2 are certainly acceptable. The method was also tested for a level of 5 µM naproxen spiked into urine, even though this was below the limit of quantification (7 µM) and this could also be found with a recovery of 98%. In addition, the membrane generally demonstrated good stability up to 10 runs with respect to these parameters. The precision based on peak area was 4.5% (relative standard deviation RSD, *n* = 10). Nonetheless, the peak area then gradually decreased and a loss of efficiency of approximately 5% was noted for the eleventh extraction in comparison to the first extraction.

## 3. Experimental

### 3.1. Chemicals, Reagents, and Sample Preparation

Naproxen, sodium hydroxide, cellulose triacetate (CTA), *o*-nitrophenyl octyl ether (NPOE), sodium perchlorate (NaClO_4_), and selectophore grade dichloromethane were obtained from Fluka (Buchs, Switzerland). Aliquat 336 (a lipophilic quaternary amine salt with a mixture of C_8_ and C_10_ chains with C_8_ predominating) was purchased from Aldrich (Milwaukee, WI, USA). Ultrapure deionized water was produced on a Nano-Pure water purification system (Barnstead, IA, USA). All reagents were of analytical grade and used without any further purification. A stock solution of naproxen at a concentration of 1 mM was prepared in methanol and was stored in the refrigerator at 10 °C. Standard solutions for studying the extraction performance were prepared by spiking solutions of 100 μM NaClO_4_ at pH 8 (adjusted with 1 M NaOH) with the naproxen to obtain a concentration of 50 μM. Drug-free urine samples were collected from healthy donors. Naproxen at different concentration levels was spiked into 300 μL of blank urine samples adjusted to pH 8. The urine samples were diluted 1:3 with acetonitrile and centrifuged at 6000× *g* (Mikro 120, Hettich AG, Bäch, Switzerland) for 10 min. The supernatant obtained was then subjected directly to analysis by the proposed method. 

### 3.2. Membrane Preparation

The membranes were prepared by casting a solution with different proportions of CTA as base polymer, NPOE as plasticizer, and Aliquat 336 as cationic carrier in 2 mL dichloromethane. The solution was poured and spread evenly into an 8 cm diameter glass petri-dish and the solvent was allowed to gradually evaporate overnight. Modification of the counter-ion in the membranes was achieved by immersing the prepared membranes for 24 h in 20 mL of a stirred 2 M NaClO_4_ solution. The membrane thickness was determined with a digital micrometer (MDC-1, Mitutoyo Corporation, Kawasaki, Japan).

### 3.3. Electric Field Driven Extraction System

The extraction system is based on two programmable syringe pumps (MD-1001/1020, Bioanalytical Systems, West Lafayette, IN, USA) fitted with 1 mL syringes for introduction of the donor and acceptor solutions. A Rheodyne 7125 injection valve (IDEX, Oak Harbor, WA, USA) fitted with a 10 μL loop was used for sample introduction. The extraction cell consisted of two identical PMMA plates (25 × 20 × 10 mm), each bearing a matching channel. Both donor and acceptor plates had a 15 mm long rectangular channel with a width of 1 mm, a depth of 0.1 mm, and a volume of 1.5 µL. To set up the extraction cell, the two PMMA plates were screw-clamped together with four M3 screws with a membrane (20 mm length × 3 mm width) sandwiched between them. The active membrane surface area was 15 mm^2^. Teflon PFA tubing (IDEX, Oak Harbor, WA, USA) with 0.01 in. inner diameter and 1/16 in, outer diameter was attached with ¼ in. × 28-UNF fittings to the inlets of donor and acceptor channels. Platinum wires (0.5 mm diameter) were inserted into each half of the cell as electrodes. A high voltage power supply module (CA05N, EMCO, Sutter Creek, CA, USA) was used to apply a negative voltage of 150 V at the donor side while the acceptor channel was grounded. Note that special care is required to avoid accidental exposure, such as a safety cage fitted with a microswitch to interrupt the high voltage on opening. Fused silica capillaries with 100 µm inner diameter and 365 µm outer diameter were attached with 1/4” × 28-UNF fittings to the outlets of donor and acceptor channels with the help of tubing sleeves. A UV-transparent window was created in the polyimide clad fused silica tube at the acceptor outlet at a distance of 10 cm from the cell and UV-absorption detection of naproxen at 331 nm was accomplished with a Spectra 100 UV–Vis detector (Thermo Separation Products, San Jose, CA, USA). Data were acquired and recorded with an e-corder data acquisition system (eDAQ, Denistone East, NSW, Australia).

## 4. Conclusions

The use of EME in an FIA method for the separation of an analyte from the sample matrix in order to enable selective quantification was demonstrated successfully. The determination of naproxen in urine by this method should be possible as long as no other species which shows an absorbance at the same wavelength and which can be extracted under the same conditions is ingested. The concept should prove useful beyond this application and with other quantification methods and its potential has not yet been fully explored. In particular, besides matrix elimination the method can also be employed for preconcentration when detection limits are not sufficient. Also, miniaturization was not necessary for this application. The new further scaled down EME cell has now donor and acceptor chamber volumes of only 1.5 µL. When quantification methods are employed for which this volume is not a limit, preconcentration from relatively small total sample volumes are possible.

## Figures and Tables

**Table 1:** Typical operating sequence.

Step	Operation	Position of Injector Valve	Donor Flow Rate (μL/min)	Acceptor Flow Rate (μL/min)
1	Flushing of manifold	Load	2	2
2	Turning off acceptor flow	Load	2	0
3	Injecting sample into injection valve	Load	2	0
4	Introducing sample into extraction cell and turning on high voltage supply	Inject	2	0
5	Turning off high voltage supply	Inject	2	0
6	Turning on acceptor pump and purging acceptor solution through UV-detector	Inject	2	1–4
7	Flushing of manifold	Load	2	2

**Table 2:** Standard deviations and recoveries of naproxen from spiked urine samples.

Amount Added (μM)	Amount Found (μM)	Relative Recovery (%)	RSD% (*n* = 8)
200	197.0	98.5	5.5
50	49.5	99.0	4.8
